# Macro-invertebrate Biodiversity of a Coastal Prairie with Vernal Pool Habitat

**DOI:** 10.3897/BDJ.4.e6732

**Published:** 2016-04-27

**Authors:** Emile Fiesler, Tracy Drake

**Affiliations:** ‡ Bioveyda, Torrance, California, United States of America; § Manager, Madrona Marsh Preserve, Torrance, California, United States of America

**Keywords:** biological diversity, ephemeral pool, wetland, dune, upland, terrestrial, aquatic, invertebrate, Arthropoda, Hexapoda, Insecta, Lepidoptera, Odonata, Coleoptera, Hymenoptera, Diptera, Hemiptera, Heteroptera, Orthoptera, Neuroptera, Psocodea, Thysanoptera, Blattodea, Embioptera, Dermaptera, Microcoryphia, Collembola, Arachnida, Araneae, Acari, Mollusca, Gastropoda, checklist, taxonomy, classification, systematics, bio-inventory, macro-photography, Madrona Marsh Preserve, Torrance

## Abstract

**Background:**

The California Coastal Prairie has the highest biodiversity of North America's grasslands, but also has the highest percentage of urbanization. The most urbanized part of the California Coastal Prairie is its southernmost area, in Los Angeles County. This southernmost region, known as the Los Angeles Coastal Prairie, was historically dotted with vernal pools, and has a unique biodiverse composition. More than 99.5% of its estimated original 95 km^2^ (23,475 acres), as well as almost all its vernal pool complexes, have been lost to urbanization.

The Madrona Marsh Preserve, in Torrance, California, safeguards approximately 18 hectares (44 acres) of Los Angeles Coastal Prairie and includes a complex of vernal pools. Its aquatic biodiversity had been studied, predominantly to genus level, but its terrestrial macro-invertebrates were virtually unknown, aside from butterfly, dragonfly, and damselfly observations.

**New information:**

In order to better understand the biodiversity at the Madrona Marsh Preserve, a minimally-invasive macro-invertebrate inventory was conducted. The results of this inventory, with 689 invertebrate organisms recorded, covering eight phyla, 13 classes, 39 orders, and 222 families, are presented in this document.

## Introduction

The California Coastal Prairie has the highest biodiversity of North America's grasslands Stromberg et al. 2002, but also has the highest proportion of urbanization Loveland and Hutcheson 1995. The most urbanized part of the California Coastal Prairie is its southernmost area, located in Los Angeles County. This southernmost region, known as the Los Angeles Coastal Prairie, was historically sequined with vernal pools, and has a unique biodiverse composition Mattoni and Longcore 1997. More than 99.5% of the estimated 95 km^2^ (23,475 acres), and almost all vernal pool complexes, have been lost to urbanization Mattoni et al. 2000. The remaining estimated thirty hectares (approximately 74 acres) has undergone considerable degradation. Notwithstanding these urbanization pressures, the remaining fragments of the Los Angeles Coastal Prairie harbor a rich and understudied macro-invertebrate biodiversity.

**Figure 1.**
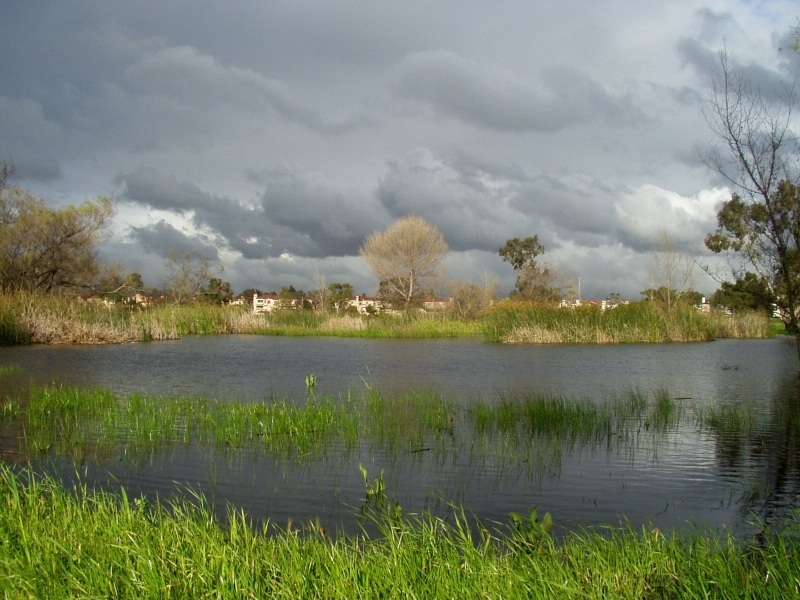
Madrona Marsh Preserve

**Figure 2.**
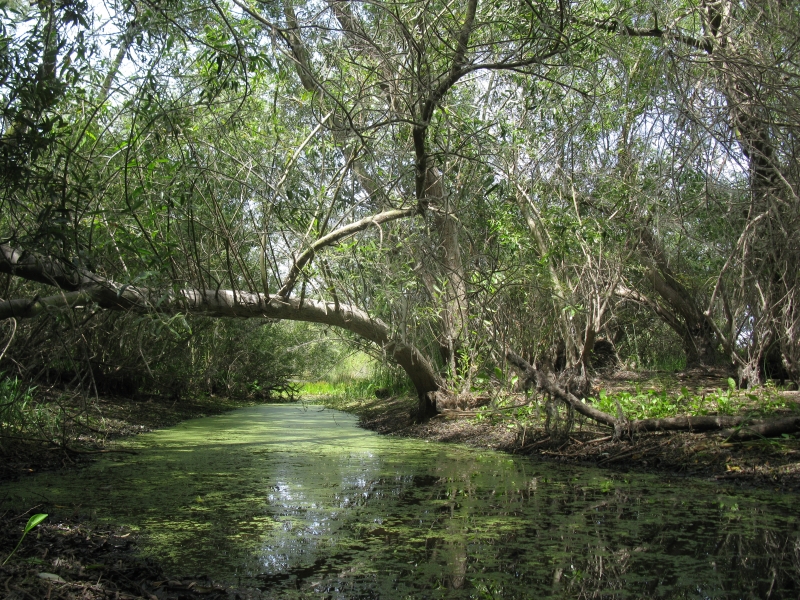
Madrona Marsh Preserve - Willow forest

The Madrona Marsh Preserve FoMM 2015 (MMP, or "the Preserve") is a public nature preserve located in the City of Torrance, California; see Figs 1, 2. At approximately 18 hectares (44 acres), it safeguards the largest remnant of Los Angeles Coastal Prairie, as well as its last functional vernal pool complex.

**Figure 3.**
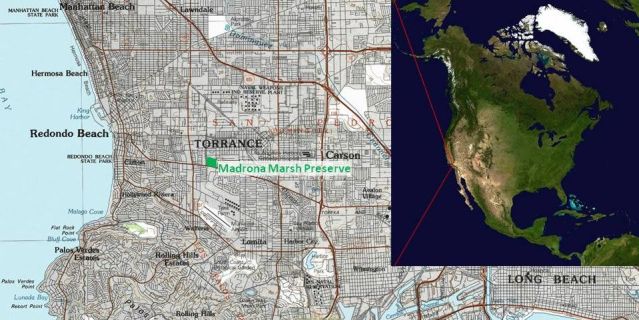
Location of Madrona Marsh Preserve (MMP) in Torrance, California. Insets depict MMP's location with respect to the United States of America and southern Los Angeles County respectively.

MMP is located in the South Bay area of Los Angeles County (33.827° North; 118.341° West) at approximately 24 meters (80 feet) elevation above sea level (see maps in Fig. 3​). The Preserve is surrounded by urban areas, including California's second largest shopping mall on its west side. It is bounded by Madrona Avenue on the west, Sepulveda Boulevard on the south, Maple Avenue on the east, and Plaza del Amo on the north. MMP is 4.2 kilometers (2.63 miles) inland from the Pacific Ocean coast and 4.8 km (3 miles) north of natural open space on the Palos Verdes Peninsula, particularly the Linden H. Chandler Preserve, managed by the Palos Verdes Peninsula Land Conservancy PVPLC 2015. It is 5.3 km (3.3 miles) west-northwest of the Bixby Marshland LACSD 2015, 6.8 km (4.2 miles) southwest of the Gardena Wil­lows Wetland Preserve Friends of Gardena Willows 2015, and 13.8 km (8.6 miles) southeast of the LAX Dunes LAWA 2015. MMP is 17.1 km (10.6 miles) southeast of the Ballona Wetlands Friends of Ballona Wetlands 2015, which includes estuarine, brackish, and freshwater marshlands, and is located scarcely outside the northern historical boundary of the Los Angeles Coastal Prairie. The Santa Monica Mountains National Recreation Area SMMNRA 2014 lies 30 km (19 miles) to the northwest, and the Channel Islands National Park NPS 2015, with Santa Barbara Island, 72 km (45 miles) to the southwest.

MMP has a Mediterranean climate with mild, dry summers and cool, wet winters. The annual average precipitation is 37.6 centimeters (14.8 inches), which almost all falls between mid October and late March. August is usually the warmest month of the year, with an average maximum temperature of 25.9 degrees Celsius (78.7 degrees Fahrenheit). December is usually the coldest month of the year with an average minimum temperature of 7.8 °C (46.0 °F). Temperature variations between night and day vary from an average of 9 °C (17 °F) in summer to 11 °C (20 °F) in winter U.S. Climate Data 2015.

**Figure 4.**
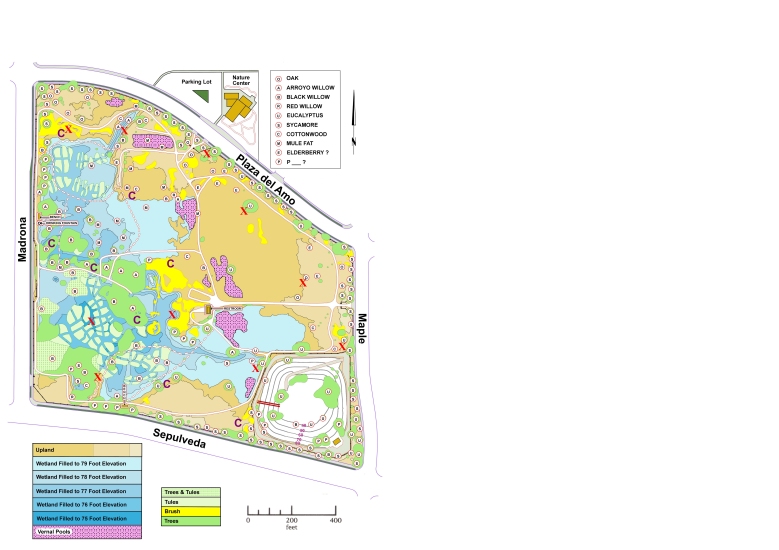
Map of Madrona Marsh Preserve. Each red X-mark indicates the location of one of the ten pitfall traps. Each purple C-mark indicates the location of one of the eight sets of three coverboards. Vegetation is shown in shades of green and yellow, vernal pools in pink, water inundation levels in shades of blue, the remaining upland areas in shades of light brown, and man-made structures in darker brown. Prominent trees and shrubs are indicated by black letters in pink-rimmed white circles. The Nature Center, surrounded by native plant gardens, is located north of Plaza del Amo, and the sump, a neighborhood runoff water collection reservoir, is located in the southeast corner of the preserve. Map created by Jerry Cole, 2011.

MMP is a nature oasis in an urban setting; see map in Fig. 4. Lo­cated in the City of Torrance, being a jewel along the Pacific Flyway Pacific_Flyway 2015, MMP is well known for its avifauna Lyle 1999, Wikipedia 2016. Aside from its well-documented bird richness, its vertebrate fauna, as well as its vascular flora, have also been studied to some extent in the past City of Torrance 1976. Although its aquatic biodiversity had been stud­ied, predominantly down to ge­nus level Angelos 2003, its ter­res­trial macro-invertebrate fauna was virtually unknown, aside from but­ter­flies and odonata observations. MMP butterfly sighting records have been primarily obtained by David Moody, Tracy Drake, Jess Morton, Martin Byhower, Kevin Larson, and Mitch Heindel. MMP odonata observations began in 2004, initiated by the second author. Summaries of the butterfly and odonata tallies have been published periodically by the second author in the *Marsh Mailing*, the periodic newsletter of the Friends of Madrona Marsh FoMM 2015.

Between November 16, 2009 and August 31st, 2011 the first author conducted a minimally-invasive bio­diversity inventory of the macro-invertebrates of the Preserve, often accompanied by the second author during field work. This study was part of a biological inventory that also included vertebrate animals and vascular plants. Goals of the bio­inventory included researching his­tor­i­cal data, providing a baseline inventory of the current MMP taxa to be able to compare future in­ven­tory date to, creating factual knowl­edge for education and outreach, as well as providing habitat restoration and enhancement recommendations for the Preserve.

The MMP macro-invertebrate bioinventory is unique in several aspects. It is the first macro-invertebrate bioinventory of its kind and scope, uniquely based on photographic vouchers, rather than the traditional captured specimen vouchers. It was performed with minimal manpower, and its scope is broad, aiming to include all taxa. This novel approach is made possible due to the advent of high-quality digital photography, which allows for taking, processing, organizing, and archiving large amounts of detailed photos, needed for such a project. The Internet is another technology that enables small teams to complete macro-invertebrates bioinventory projects in a limited time. BugGuide in specific is an extensive on-line resource with an unprecedented collection of more than 820,000 arthropod photos, taxonomically classified and organized by more than 280 editors and experts BugGuide 2014.

**Related studies by others - an overview**

Biodiversity, including urban biodiversity, is critical to human sustainability Kim and Byrne 2006. Most biodiversity studies include vertebrate animals or vascular plants, or both. Few inventories include invertebrate animals, non-vascular plants, or organisms of other kingdoms in their scope [Bibr B2997678][Bibr B2997751][Bibr B2982668]. Macro-invertebrates are ubiquitous and account for the largest percentage of all metazoan biodiversity. They also account for the largest percentage of endemic taxa and taxa of concern (threatened, endangered, or otherwise imperiled), although most such invertebrates have not yet been officially recognized as being imperiled. The International Union for the Conservation of Nature’s Red List IUCN Red List 2016 estimates the global total at about 1.3 million invertebrate species and the percentage at risk of extinction at about thirty percent (30%) of species evaluated Center for Biological Diversity 2015. Macro-invertebrates are primary candidates for monitoring environmental impacts, as they respond quickly to environmental changes due to their short life cycle. Moreover, they are a crucial building block of the food-chain, and our global biosphere in general. The famous American myrmecologist Edward O. Wilson said: "*If all mankind were to disappear*, *the world would regenerate back to the rich state of equilibrium that existed ten thousand years ago. If insects were to vanish, the environment would collapse into chaos*." Jarski 2007.

Surprisingly few macro-invertebrate or all-taxa inventories have taken place in general Kim and Byrne 2006, including in well-populated Southern California. Some prominent bio-inventories performed in Southern California that focused on, or included, macro-invertebrates, are listed in Suppl. material 1. The table, which is ordered by number of taxa found, includes studies whose results were published by the end of the MMP bioinventory in 2011. More recent studies show more up-to-date results. The bio-inventories listed in Suppl. material 1 are also briefly discussed below; ordered in decreasing proximity, or relevance, to MMP.

Mark Angelos performed a high-quality study of MMP's vernal pool arthropods, gastropods, vertebrates, and plants, between 1993 and 2003. At that time he was Research Associate in Invertebrate Zoology at the Los Angeles County of Natural History. He predominantly used nets and recorded 76 animal taxa, of which 67 arthropods, five mollusks, and four vertebrates; most of which he identified to genus Angelos 2003. Between 1997 and 2003 he performed a study of MMP's ostracods, small aquatic crustaceans, of which he found seven species in MMP’s vernal pools Angelos 2005.

W. Dwight Pierce, together with Dorothy Pool and others, performed field studies in the El Segundo Dunes from 1938 to 1939 and published highlights of their findings in a series of articles published between 1938 and 1947 Pierce et al. 1947. Their field notes, which include 187 arthropods and 38 (mostly marine) mollusks, were never published Pierce and Pool 1939.

Rudi Mattoni and his team performed a study of the flora and fauna of the 746 hectares (302 acre) El Segundo dune remnant at the west side of Los Angeles International Airport (LAX) from June 1987 to June 1988 Mattoni 1990. The used a combination of pitfall and yellow-pan traps, aerial net sweeping, beat-sheets, and Malaise trapping, as well as a mercury vapor and a ultra-violet (UV) light for night data collection. Their faunal study included invertebrates and yielded 728 arthropod and three mollusk taxa. Sand-obligate invertebrates restricted to Southern California coastal dunes were listed in his 1993 publication on the Los Angeles Coastal Prairie Mattoni 1993. A follow-up study was performed from 1992 to 1993 to sample arthropods on, and adjacent to, the El Segundo Dunes Mattoni et al. 2000. They collected 169 species in yellow pan traps and 189 species in pitfall traps. Nine (9) arthropod species (one spider, four moths, one cricket, and three weevils) were listed as endemic to the El Segundo dunes Mattoni et al. 2000.

Travis Longcore, who participated in the arthropod study mentioned above Mattoni et al. 2000, performed a five year terrestrial arthropod study of thirteen (13) locations on the Palos Verdes Peninsula, in south­western Los Angeles County, plus two locations in Crystal Cove State Park in coastal Orange County Longcore 1999. He found 204 arthropod taxa in his pitfall traps.

The Ballona Wetlands, located north of the northern boundary of the Los Angeles Prairie, consisted of approximately two thousand acres of coastal wetland habitat. Most of these wetlands have been lost to urbanization, including nine hundred acres for the construction of a small craft harbor and adjoining community in the 1960's, now known as Marina del Rey. The remaining six hundred acres consist of estuarine, brackish, and freshwater marshes, seasonal wetlands and other riparian habitats, as well as uplands. From 1980 to 1981, a one year biological study was performed on the Ballona Wetlands, which included the recording of 475 insects and mollusks Schreiber 1981. They used a combination of aerial, sweep, and aquatic nets, baited pitfall traps, Malaise trap, yellow pan traps, UV light traps, soil sifting, and Berlese funnel sifting. The specimens have been absorbed into the main entomology collection at the Los Angeles County Museum of Natural History.

Emile Fiesler conducted a macroinvertebrate bioinventory of the Oxford Basin in 2010. The Oxford Basin, located in Marina del Rey, is one of the last remaining areas with intertidal mud flat habitat in Los Angeles County. He recorded 85 terrestrial and marine taxa (two mollusks, eight spiders, 67 insects, and eight other arthropods), using macro-photography, during three half days of field work spread over four months Fiesler 2010.

Louis LaPierre and Pamela Wright surveyed the arthropod fauna of the Kenneth Hahn State Recreation Area during June and July of 2000 LaPierre and Wright 2000. The Kenneth Hahn State Recreation Area is located in the Baldwin Hills, which are situated approximately halfway between the Ballona Wetlands near the Pacific Ocean and downtown Los Angeles. They classified the habitat as "degraded Coastal Sage Scrub, dominated by California Sagebrush and Coyote Brush." The methods they employed were: pitfall traps, Malaise traps, yellow pan traps, beating sheets, aerial nets, black-lights and a mercury vapor lamp. They found 114 arthropod taxa (fourteen spiders, two isopods, 70 Lepidoptera (33 butterflies and 37 moths), 15 Coleoptera, 7 Hymenoptera, and six other insects) plus more than 99 undetermined morphospecies.

Emile Fiesler conducted a study of the non-marine macro-invertebrates of the Santa Monica Mountains National Recreation Area (SMMNRA) Fiesler 2009. This pilot study consisted in estimating the num­ber of non-marine macro-invertebrate families present in SMMNRA, compiling a complete list of butterflies that could potentially be found in the SMMNRA, compiling a list of invertebrates of concern (rare, endemic, threatened, or endangered), and creating a preliminary list of 160 non-marine macro-invertebrates representing 160 distinct families. The family list is comprised of twenty arachnids, seven myriapods, three crustaceans, two mollusks, one nematomorph, and the rest hexapods. He extracted this list from 410 (pre-project) plus 530 (new) specimens that are vouchered by approximately 6,200 photos. Based on this pilot study, the first author created an on-line field guide with the title: "Field Guide to the Insects and other Invertebrates of the Santa Monica Mountains" Fiesler 2016.

Peter Bryant has set-up an extensive internet website on the biodiversity of "Orange County and nearby places" Bryant 2014. Orange County is situated directly south and southeast of Los Angeles County. The hierarchically organized website's scope includes all taxa: animals, plants, fungi, and more. Each taxon is usually represented by its common name, its scientific name, and one or more photos. Each photo has a caption, which typically contains information on the photographer and the location and date where the photo was taken. Selected taxa are accompanied by information on biology, ecology, taxonomy, or other topics.

Nancy Hamlett is in charge of an all-taxa biota study of the 210 ha (85 acre) Robert J. Bernard Biological Field Station of the Claremont Colleges. The Bernard Field Station is located in the city of Claremont, approximately 58 km (36 miles) east of downtown Los Angeles. The ongoing study covered 352 invertebrate taxa, as well as vertebrates, plants, and lichens Hamlett 2014.

Studies on plants, vertebrate animals, and insects of the 2,469 ha (6,100 acre) Philip L. Boyd Deep Canyon Desert Natural Reserve have been coordinated by the University of California, Riverside. The Reserve is located five miles south of the City of Palm Desert in Riverside County; in the Colorado Subdivision of the Sonoran Desert. A succession of researchers, students, and visiting scientists have surveyed insects at the Reserve over the past 34 years Frommer 1988. The insect survey, primarily utilizing Malaise traps, has yielded about 5,000 insect taxa, 2,600 of which have been classified. The specimens are curated at the University of California, Riverside entomology collection.

An all-taxa bioinventory was conducted as part of the San Diego Bay Integrated Natural Resources Management Plan TD 2011. A key finding was that climate change and invasive species are now principal drivers of change, whereas habitat loss due to development was the main driver in the past for the San Diego Bay. More than 650 taxa of marine, estuarine, and salt marsh invertebrates were recorded at species-, or genus-level, as part of the bioinventory that was primarily based on trawling.

Gordon Pratt conducted a survey of the terrestrial arthropods of Edwards Air Force Base between January 1996 and October 1998 Pratt 2000. Edwards Air Force Base is located in the Mojave Desert, in northeastern Los Angeles County, approximately 36 km (22 miles) northeast of Lancaster. He encountered 1,536 species from 32 sites, using sight, nets, and pitfall traps.

The Southern California Association of Marine Invertebrate Taxonomists (SCAMIT) has compiled a list of benthic macro- and mega-invertebrates. The data is compiled from various infaunal and epifaunal monitoring and research programs conducted in the Southern California Bight, mostly based on trawling. The Southern California Bight area covered by the list extends from Point Conception in Santa Barbara County, California to Bahía Todos Santos, Baja California Sur, Mexico, in intertidal to 1000 meter depths. This geographical area includes the off-shore areas of Los Angeles, Orange, San Diego, and Ventura Counties, plus the southern section of Santa Barbara County and the northwestern-most section of Baja California Norte. The ninth edition lists 3,177 taxa Cadien et al. 2014.

## Material and methods

The following sections detail the methodologies used for the terrestrial and aquatic invertebrates surveys. The surveys performed for this project are unique as they were conducted with minimal invasiveness. A minimally invasive study aims at studying organisms with minimum disturbance and without collecting them; relying primarily on high-resolution digital photography. To maximize taxonomic identification success, close-up photos are taken from various angles, where possible. Nevertheless, not having a specimen in hand tends to increase the complexity of taxonomic identification, as certain characteristic details, some of which could be internal, might not be visible.

### Terrestrial invertebrates data collection methodology

Common terrestrial invertebrates are mostly comprised of hexapods and arachnids. Other, less species-rich taxa include: isopods, terrestrial snails, and earthworms. Terrestrial invertebrates can be divided into (1) herbivores and detritivores, which are the primary consumers, and (2) predators, parasites, and parasitoids. The herbivores and detritivores comprise the lower levels of the food chain. Terrestrial herbivores are usually associated with certain host- or food-plants, which are predominantly plants native to the area. Native plants are therefore the base for a healthy terrestrial ecosystem.

The minimally invasive terrestrial invertebrate survey was performed in stages, using the following methodologies:

- visual detection and photo-documenting salient organisms, as well as evidence of their presence;
- periodic coverboard inspection;
- overnight pitfall trapping focused on flightless invertebrates; and
- night light collection, using both ultraviolet and broad-spectrum incandescent illumination

Each of these four data collection methodologies is explained below.

Visual detection took place during all visits of the site and formed an important part of the data collection. We made an effort to choose a different transect for every visit to maximize coverage of the area. We surveyed at various parts of the day, from the early morning to after dark, with an average of 2.5 hours per survey. Keen visual observation enables recording taxa that are not typically obtained by other methods and would go unnoticed. Specimens were photographed using a Canon Powershot A650 IS in macro-photography setting. Each specimen was photographed from various angles, aiming for dorsal, lateral, and frontal exposures; limited by the time the specimen remained present.

Eight sets of three wooden coverboards were placed throughout the preserve, mostly at shady locations to minimize direct sun exposure. Each of the 24 square coverboards measured three by three feet (91 centimeters) in length and 0.5" inch (1.3 cm) thick. The map in Fig. 4 contains the locations of the coverboards. During each of the transects followed during the visual surveys explained above, we would examine the coverboards that we encountered en route. One person would quickly lift the coverboard in such a way as to maximize exposure of sunlight of the area that had been covered by the board. Specimens encountered would be photographed in a manner similar to that explained above.

Ten independent pitfall traps, consisting of 1.75-liter white polypropylene containers with smooth, near-vertical walls, were placed on June 17 and September 10, 2010, at various well-spaced locations from the waterline to the higher parts of the core upland areas Fig. 4. The containers were placed in holes dug into the soil such that their upper rims were flush with the surrounding ground. The locations were chosen to maximize inclusion of habitats. Some were placed under the canopy of shrubs and trees, like western sycamore (*Platanus racemosa* Nutt.), coast live oak (*Quercus agrifolia* Née), and Mexican elderberry (Sambucus nigra L. subsp. caerulea (Raf.) Bolli), to catch animals that might fall down due to the wind or other mechanisms. The next morning the pitfall traps were collected, the holes closed, and their content documented.

The night light data collection took place on April 13, May 12, June 17, August 10, September 9, and December 1 of 2010, and on February 23, April 6, August 23, and November 8, 2011; mostly on dates close to a new moon. New moon assures minimum light from the sky as well as maximizes insect, especially moth, activity, apparently due to high light polarization levels Nowinszky and Puskás 2012. At night, we used ultraviolet light stations, as well as broad-spectrum incandescent (“white”) light stations. Given the need for powering the lights, we typically deployed at least one ultraviolet station in the central area of the Preserve, where we used the available 110 Volt AC outlet and multiple extension cords to reach places near the water or on some of the walking trails. The stations themselves consist of a light colored sheet draped over a construction made from ladders, poles, and wooden boards. Another ultraviolet light station was created by draping a light colored sheet over the hood or trunk of a car. This light was powered by the 12 DC car outlet. A “white” light station was usually a battery-powered lamp placed on a light colored sheet on the ground. We typically chose a location on a trail due to the absence of vegetation for spreading out the sheet, as well as to optimize our access. The location on open trail areas is also likely to intercept walkways of ground-dwelling invertebrates. Once a specimen settles onto one of the sheets, we recorded it photographically. We usually had a team of assistants helping with setup of the stations as well as help the authors keeping track of new specimen arrivals.

The combined results of all the data collection methods described above, consists of more than 21,000 digital photos. The photos have been stored in an hierarchical data structure ordered by year and date, based on each photo's date and time stamp. Representatives of each specimen photographed have been copied into a separate hierarchical data structure, sorted on taxonomic order. Each digital photo in the taxonomic data structure has been assigned a filename that includes the organism's scientific name and common name, together with other available data, including size and field-marks. This file naming strategy enables fast, operating-system-level, text-based searches for retrieving data.

### Aquatic invertebrates data collection methodology

In addition to the data collection methodologies described above, we also performed dedicated sampling of MMP's aquatic habitats. Data collection of aquatic organisms was performed several times a month between April and July 2010, using a combination of two techniques:

1. ***Acute sampling without a net***: The bulb of a large baster was fully squeezed before inserting the tip into the water. The tip of the baster was moved around slowly; slowly releasing the bulb to collect the sample. The resulting sample was slowly squeezed out into a sieve with a mesh size of about 0.1 mm. This was repeated several times until the sieve became relatively full. The sieve was then turned upside down over a collection container, which usually was a small glass jar, and some clear water was squeezed through the other side of the sieve, releasing the trapped content into the jar. This process was repeated until a sample of sufficient volume had been collected.
2. ***Large quantity sampling with a net***: First, about four ounces of water were collected and poured into a small container. Next, a rectangular, four-by-three inch net, with approximately 1 mm mesh size, was swished back-and-forth through the water, collecting whatever is in the water. Once the net had a full sample, it was pulled out of the water. Large debris, such as pieces of *Schoenoplectus californicus* (C. A. Mey.) Soják (California Tule) and other plant detritus, was removed manually and the net subsequently turned inside out into the container. The sample in the net would free itself into the water in the basin by moving the net through the water. Holding the container at an angle helps to keep the water in one relatively small location. This was repeated several times until a sample of sufficient volume had been collected. The resulting content was poured into a collection jar.

These techniques were applied at a variety of locations, including small vernal pools and the sump, which is a large basin that collects runoff water from the surrounding neighborhood. The sampling locations were changed each week, and at these locations various micro-habitats (open water, near emergent vegetation, near tree roots, and under floating aquatic vegetation) were sampled at varying depths (from the shallowest to the deepest water), to maximize the chances of capturing organisms from a broad range of habitats.

The samples were taken to the MMP Nature Center’s curation lab, where they were examined by the first author, using a variety of microscopes and photographically recorded using a number of digital imaging techniques. After examination, the samples were returned to the Preserve. The results of the aquatic sampling have been integrated into Suppl. material 2.

### Arthropod taxonomy

As the number of described macro-invertebrate taxa is immense, estimated at more than 1,300,000 Current Results 2015, and its classification in constant flux, a continuously updated on-line resource was used for taxonomic hierarchy and nomenclature. The overwhelming majority of the macro-invertebrate taxa recorded during the MMP bioinventory are arthropods. BugGuide BugGuide 2014 offers a taxonomy for arthropods that is updated using the latest information by consent of the scientific community directly, or indirectly, contributing to the site. The information is stored in a hierarchical taxonomic way and is publicly available. The arthropod taxonomy presented in BugGuide, for the 2009 to 2012 period of the MMP bioinventory, is used in this publication.

## Results

Before presenting the overall results, the outcome of a few selected data collection methodologies are presented, as they tend to contain method-specific data of interest.

### Pitfall trap results

**Figure 5.**
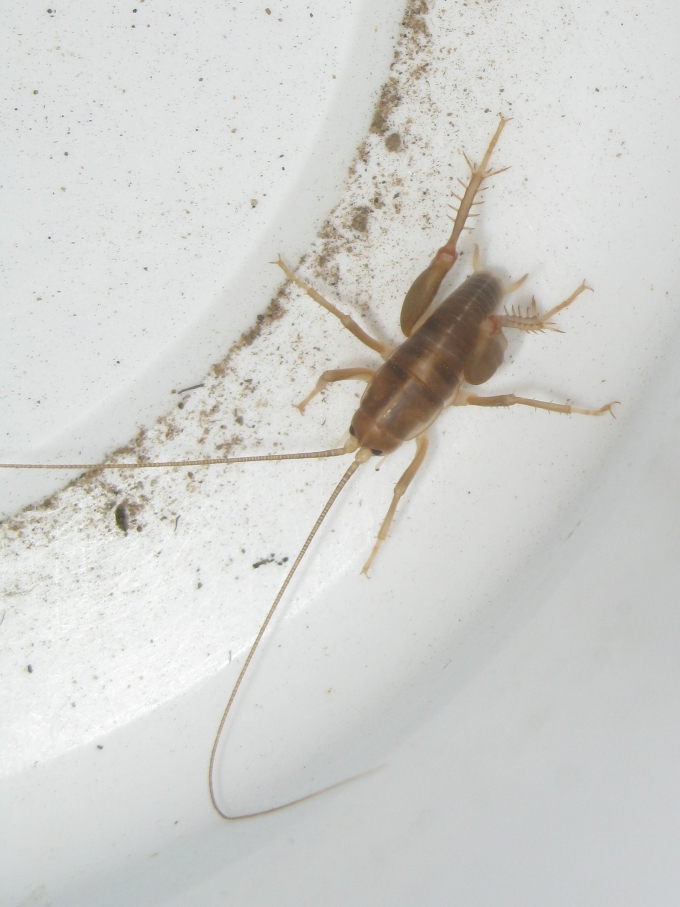
*Ceuthophilus hesperus clunicornis* Hubbell, 1936 (San Diego camel cricket)

The pitfall trap results yielded several species not recorded by the other methodologies. The most striking find was *Ceuthophilus hesperus clunicornis* Hubbell, 1936, (San Diego camel cricket); see Fig. 5. Given the fact that they are flightless, and hence limited in their dispersal, it is very likely that camel crickets have been present at the site for a long time. Camel crickets are among the least studied orthopterans (order: Orthoptera, or “straight-winged” insects), which include all grass­hoppers, katydids, crickets, and allies.

**Figure 6.**
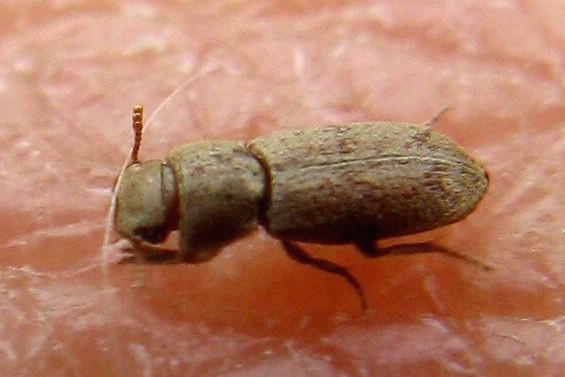
*Lepidocnemeplatia sericea* (Horn, 1870) (a darkling beetle)

**Figure 7.**
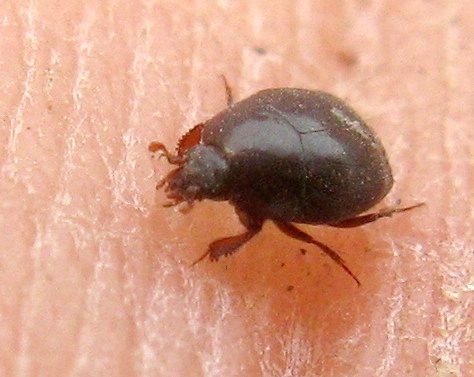
*Xerosaprinus* sp. (a clown beetle)

**Figure 8.**
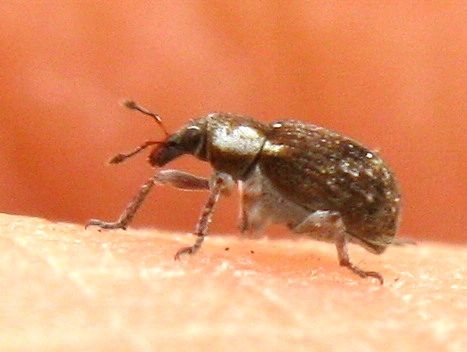
*Stenopelmus rufinasus* Gyllenhal, 1835 (Azolla weevil)

Other unique pitfall trap discoveries are three small native beetles:

- A somewhat elongated beige-brown beetle with two unusually long claws on each foreleg. This is *Lepidocnemeplatia sericea* (Horn, 1870), a darkling beetle, see Fig. 6, (family: Tene­brionidae); which is fairly widespread in the Southwestern United States, but not often encountered.
- A new family for the Preserve was the discovery of *Xerosaprinus* sp., a clown beetle, see Fig. 7, in the family Histeridae.
- The third beetle was a snout beetle, also known as a weevil. It was in one of the two traps near the waterline. It is *Stenopelmus rufinasus* Gyllenhal, 1835, also known as Azolla weevil or water fern weevil; see Fig. 8​. It's life cycle is dependent on the presence of the native *Azolla filiculoides* Lam. (Pacific mosquito fern) in the family Azollaceae.

**Figure 9.**
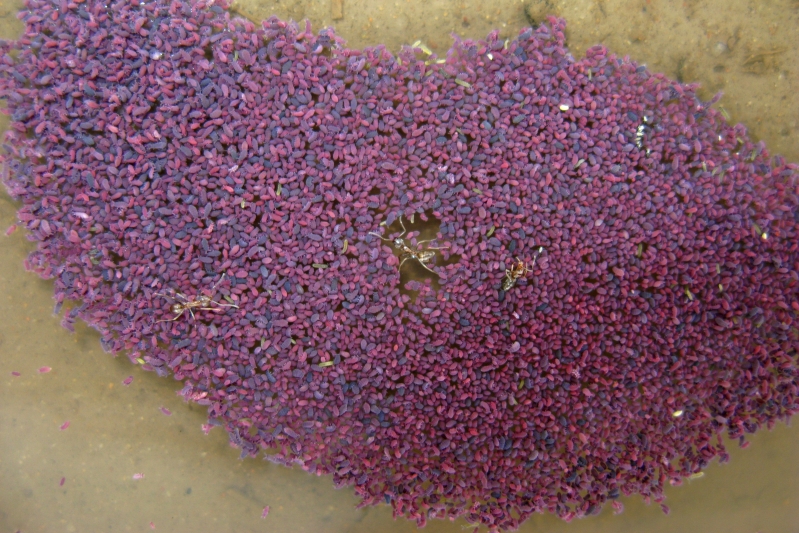
Collembola (Springtails) and *Linepithema humile* (Mayr, 1868) (Argentine Ants)

**Figure 10.**
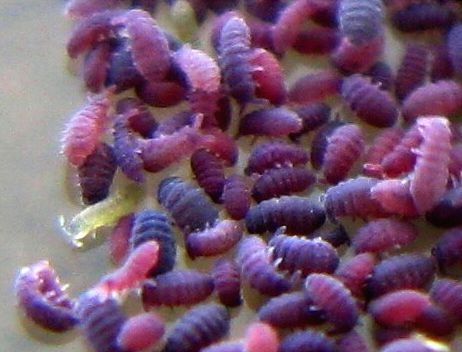
Collembola (Springtails) and *Linepithema humile* (Mayr, 1868) (Argentine Ants) - detail

The pitfall trap closest to the waterline yielded, besides two aquatic ground beetles, many hundreds of springtails (class: Collembola); see Figs 9, 10. The data collected during the pitfall trap sessions have been added to the taxon list in Suppl. material 2.

### Nighttime observations

An overview of the results of our night data collection efforts are shown in Table 1.

The results of the night data col­lection show that moths are by far the most frequently encountered, followed by chironomidae (non-biting midges) and various other Nematocera ("lower flies"). The maximum number of specimens were encoun­tered in May, followed by June and September. Only five indi­vid­uals, and no moths at all, were recorded for December. The spe­cies recorded by our night data collection efforts are included in Suppl. material 2.

### Aquatic invertebrates

Besides macro-invertebrates, we also recorded the presence of some of the more salient aquatic micro-invertebrates that were caught in our water samples. These taxa have been added to Suppl. material 2, but likely represent only a small fraction of MMP's micro-fauna.

It is difficult to compare our data one-to-one with those obtained by Mark Angelos (1993–2004; as detailed above), due to the fact that his specimens were identified to genus or family level and those in our study (2009–2010) to various levels ranging from family to (sub)species. Nevertheless, we attempt to compare our data by estimating the number of genera obtained in each study, and then comparing which genera are present in both studies. Genera recorded during 2009–2010, but not during 1993–2004, are added in the column labeled "gained", and genera recorded during 1993–2004, but not during 2009–2010, are added in a column labeled "lost" The combined result is shown in Table 2.

It is difficult to proof that an organism is not present in a given area. There are however some salient organisms that have not been encountered during our study or reported during the last decade. We are fairly sure that these organisms have been extirpated, or become extinct, at MMP. The following two salient invertebrate taxa were recorded by Mark Angelos and have not been observed during the last decade: *Lestes congener* Hagen, 1861 (Spotted Spreadwing) and *Mesovelia* sp. (Water Treader). These losses might be due to a decrease in water quality. Also potentially extirpated are *Buenoa* and *Nononecta* spp. (Backswimmers), *Haliplus* sp. (Crawling Water Beetle), Hydraenidae (Minute Moss Beetles), and Noteridae (Burrowing Water Beetles); although more intensive aquatic sampling over a longer time frame might reveal the continued presence of these taxa.

Mark Angelos has donated his MMP aquatic invertebrate specimen collection, and associated documentation, to the MMP Nature Center. This enables the possibility to identify his specimens to species level and recreate a detailed MMP aquatic invertebrate fauna for the period 1993–2004, as well as a higher quality comparison to MMP's current aquatic invertebrate fauna.

### Environmental conditions

To put the data collection period in perspective, we add some information on the weather during the timeframe of the project. A lot of the data was collected in the year 2010, which was a year with unusual weather. The spring and summer of 2010 were surprisingly breezy, and the coolest on record, temperature-wise. However, the highest temperatures ever measured in Los Angeles were recorded on September 27, 2010, with an all-time record of 113 degrees Fahrenheit for the downtown area. Furthermore, in late December 2010, Los Angeles received an exceptional amount of rain from one storm-system, more than seven inches. Invertebrates are exothermic, relying on the ambient temperature to warm their body in order to get active. The cool and breezy weather during the peak annual activity time was therefore not optimal for invertebrate abundance. During 2011 we collected data until August 30th. This year (2011) also had a cool summer. We nevertheless recorded a large amount of data, and can only expect to encounter even larger biodiversity during more 'normal' years.

## Discussion

During the project period, more than 12,500 digital photos were taken, processed, identified, and archived. Besides those, more than 9,500 digital photos had been taken and archived during the period from 2003 until the start of the project. A small fraction of this older data have been processed and identified and these taxa have been included in this report. Combining all the available data, a total of 689 invertebrate organisms were recorded, covering 8 phyla, 13 classes, 39 orders, and 222 families. A summary of the statistics for the highest, and some of the most salient, taxa are given in Table 3. The orders with the largest number of recorded taxa are, in decreasing order: Diptera (155 taxa), Coleoptera (106 taxa), Hymenoptera (101 taxa), Lepidoptera (84 taxa), Hemiptera (82 taxa), and Araneae (48 taxa).

All the 689 taxa are listed in Suppl. material 2, sorted alphabetically in a hierarchical way, starting at phylum level, down to subspecific level. The list contains all invertebrate taxa recorded, including small arthropods like aphids, thrips, and mites, as well as all recorded microscopic aquatic taxa. Some taxa above species level appear identical in Suppl. material 2. These taxa are based on sufficiently different morphotypes.

**Figure 11.**
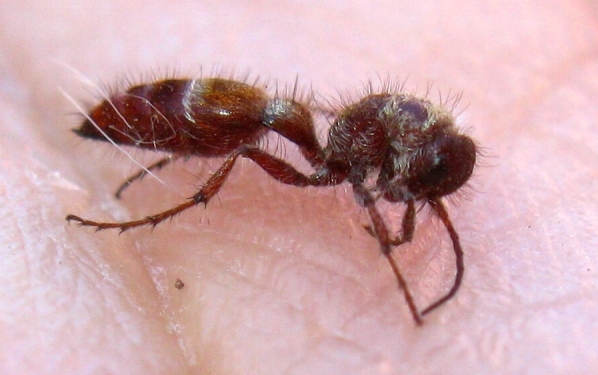
*Typhoctes peculiaris peculiaris* (Cresson, 1875) (a bradynobaenid wasp) female

**Figure 12.**
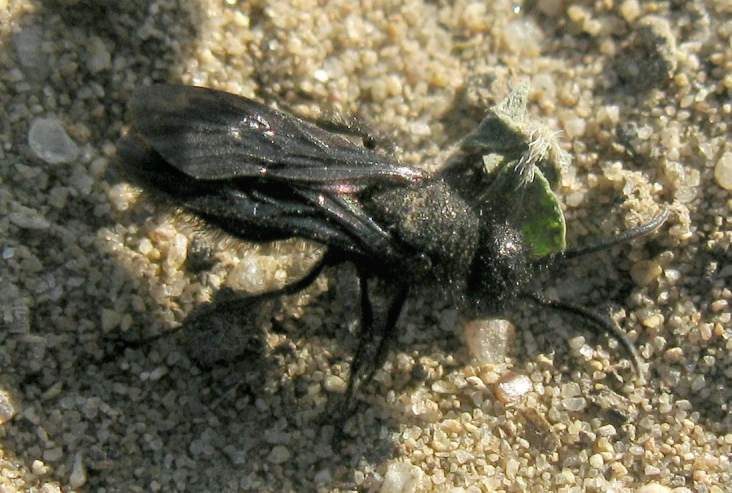
*Pseudomethoca anthracina* (Fox, 1892) (a velvet ant) male

**Figure 13.**
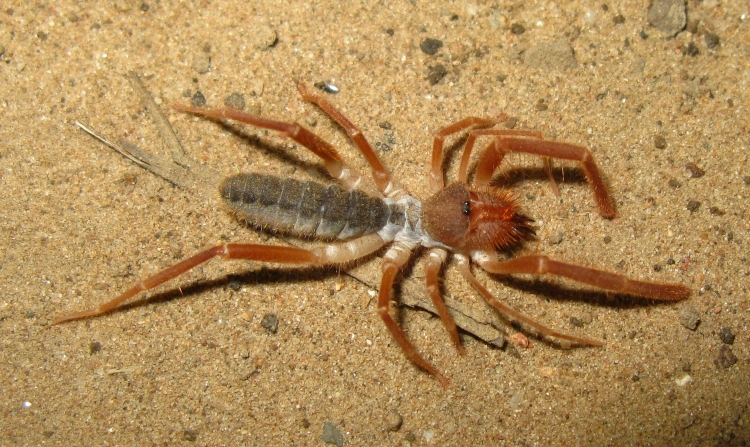
*Eremobates* sp. (a solifugid)

**Figure 14.**
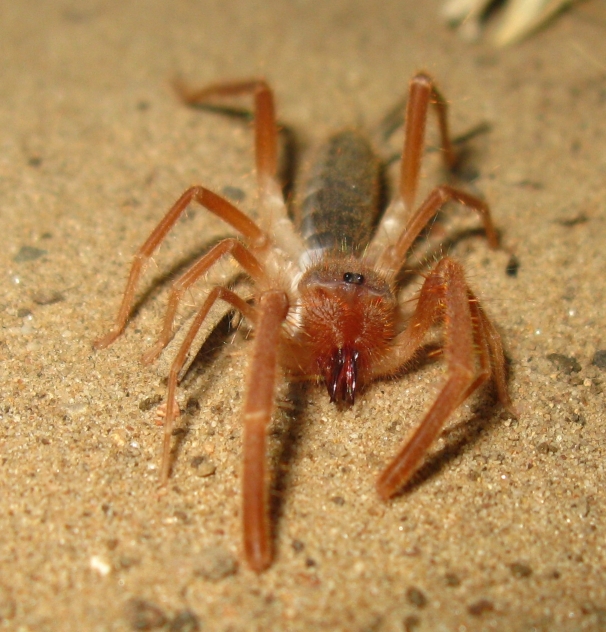
*Eremobates* sp. (a solifugid)

Of particular interest is the presence of native flightless species, that have likely persisted throughout the human-influenced history of the land that is now Madrona Marsh Preserve, including:

- *Typhoctes peculiaris peculiaris* (Cresson, 1875), a bradynobaenid wasp, see Fig. 11; whose females are flightless,
- *Pseudomethoca anthracina* (Fox, 1892), a velvet ant, see Fig. 12; whose females are also flightless,
- *Eremobates* sp., a large solifugid, see Figs 13, 14, and
- *Ceuthophilus hesperus clunicornis* Hubbell, 1936, San Diego Camel Cricket, see Fig. 5.

Some taxa were notable for their absence, including Diplopoda (millipedes), Opiliones (harvestmen), Scorpiones (scorpions), Pseudoscorpiones (pseudoscorpions), cockroaches, Mantodea (mantids), Phasmida (stick insects), Nematomorpha (hairworms), and Diplura. Diplopoda (millipedes) have been observed by Walt Wright (MMP manager preceding Tracy Drake) in the past, but have possibly been extirpated. Similarly, only one kind of Chilopoda (centipede) was found, and only in small numbers. Native Corydiidae (sand roaches) are not expected at MMP due to lack of soft sand habitat, and adventive cockroaches have been recorded in urban areas not far from the Preserve, but are closely associated with human activity and not expected to be permanent resident on the Preserve. Native mantids are relatively uncommon in Los Angeles County and not expected at MMP due to its isolation within urbanization. Adventive mantids are introduced in urban settings as pest control, and it is no surprise not to find them on the Preserve either. Most of the remaining taxa that were not found are associated with fairly undisturbed habitats or other habitats that are not present at the Preserve.

**Figure 15.**
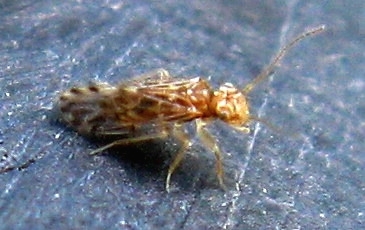
*Ectopsocus strauchi* Enderlein, 1906 (a barklouse)

**Figure 16.**
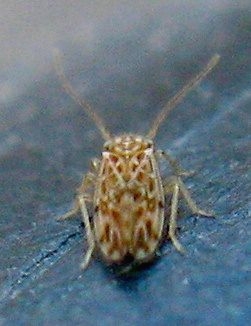
*Ectopsocus strauchi* Enderlein 1906 (a barklouse)

Some notorious and ubiquitous Los Angeles County adventives, like *Ceratitis capitata* (Wiedemann, 1824) (Mediterranean Fruit Fly), *Homalodisca vitripennis* (Germar, 1821) (Glassy-winged Sharpshooter), and *Sophonia orientalis* (Matsumura, 1912) (Oriental Leafhopper) were not recorded from MMP. An interesting adventive species that was recorded from the Preserve is the Outer Barklouse *Ectopsocus strauchi* Enderlein 1906, see Figs 15, 16, which is native to Europe and found in eastern parts of the United States. This finding constitutes the first west coast record for this species. Overall, relatively few adventive taxa were identified: six introduced for bio-control, two migrants that expanded their range, and 44 other adventive taxa, of which only a fraction might be labeled as destructive pest. All three categories of adventive taxa combined represent less than 8 % of MMP's invertebrate fauna.

No doubt species will be found in the future that are not on the list. These will predominantly be new colonizers, visiting vagrants, and obscure spe­cies in under-sampled habitats, including in­fauna of the soil, microscopic aquatic fauna, and ectoparasites of vertebrates, which were outside the scope of this project. Also, as mentioned before, years with different weather patterns are likely to reveal species that were not encountered during the study period.

This study can nevertheless be considered quite successful, given the available time and other resources. The list of recorded taxa will change over time, as some species come and some go, especially winged ones. The current list constitutes a solid baseline to compare future data with, and can be used as data source for educational and outreach purposes.

## Supplementary Material

Supplementary material 1 Supplementary file 1 = Selected biological surveys and inventories, that include macro-invertebrates, for Southern California Data type: data spreadsheet
Brief description: The county, or counties, where the survey took place is highlighted in light green for surveys areas including Los Angeles County.
Abbreviations and symbols used: A = arachnids & myriapods; B = Beach; F = fresh-water invertebrates; H = hexapods (= insects and kin); ha = hectares; invert. = invertebrates; LA = Los Angeles County; LAX = Los Angeles International Airport; "n/a" (non-applicable; highlighted in orange) = survey range does not include this habitat; M = mollusks; MI = marine invertebrates; OC = Orange County; PV = Palos Verdes Peninsula; SD = San Diego County; SRA = State Recreation Area; "unk." = unknown; "~" = approximately; ">" = greater than; "+" = at least.
File: oo_82014.jpg E. Fiesler

Supplementary material 2 Supplementary file 2 = Annotated taxonomic List of MMP Invertebrates Data type: taxa checklist
Brief description: The following is a list of all invertebrates recorded at Madrona Marsh Preserve for the project described in this document.
For each taxon, the columnar data in table in the Appendix consists of:
Scientific name & taxonomy: Phylum, Class, Order, Family, Tribe, Genus, Species, Subspecies / Variant
Common Name
Whether the taxon is adventive / non-native
Whether the specimen was observed at night
Abbreviations used: A = adventive taxon; B = introduced for biocontrol; cf. = (Latin: *confer*) = compare; E = migrant that expanded its range; imm = immature; N = recorded after sunset; n/a = not applicable; nr. = near; S. Cal. = Southern California; s.l. = (Latin: *sensu lato*) = broadly; var. = variant.
Color coding used: GREEN = high taxonomic certainty; YELLOW = intermediate taxonomic certainty; ORANGE = low taxonomic certainty.
File: oo_82016.xls E. Fiesler

## Figures and Tables

**Table 1.** Overview tally of specimens recorded during night data collection sessions, grouped by selected higher taxa (order, superfamily, family, etc.). Related higher taxa are grouped together and sorted in phylogenic order Wheeler et al. 2001. Higher taxon groups have alternating background coloration to aid readability. Note: Numbers in boldface font indicate maxima - greater than 1 - for each taxon. (Sub)totals per taxon, and per month, are added in the margins.

			**Year:**		**2010**						**2011**				
**Phylum**	**Class**	**Order**	**Taxon group**	Common name	**Apr**	**May**	**Jun**	**Aug**	**Sep**	**Dec**	**Feb**	**Apr**	**Aug**	**Nov**	**Total**
Annelida	Clite-llata	Haplotaxida	Haplotaxida	Earthworms & kin								1			1
Artho-poda	Arachnida		Arachnida	Spiders & kin	1	2	1	**3**	1	2	1		2		13
	Collembola		Collembola	Springtails		**3**	2	2	1				1		9
	Insecta	Orthoptera	Orthoptera	Grasshoppers & kin								1			1
		Dermaptera	Dermaptera	Earwigs		1									1
		Hemiptera	Aphidoidea	Aphids	1	1	1						1		4
			Auchenorrhyncha	Hoppers		2	4	3	**6**				2	1	18
			Psylloidea	Psyllids				1	1						2
			Heteroptera	Bugs			1	1					1		3
		Psocoptera	Psocoptera	Barklice	1	1							**2**		4
		Coleoptera	Scarabeidae	Scarabs		4	**5**		1			1			11
			other Coleoptera	other Beetles		1	**3**	**3**	2			1	6		16
		Neuroptera	Hemerobiiformia	Lacewings					1						1
		Hymenoptera	"Parasitica"	Parasitoid Wasps		1		1	1				**3**	1	7
			Formicidae	Ants				**2**	1				1		4
		Lepidoptera	<no taxon>	Moths	3	**33**	24	11	12		2	5	16	4	**110**
		Diptera	Chironomidae	Midges	6	**15**	4		1			2			28
			other Nematocera	other "lower" Flies	**9**	5	3		8	2			1	2	30
			Brachycera	"higher" Flies	**4**	2	1	3	2			3	3	1	19
			**Total:**		25	**71**	49	30	38	4	3	14	39	9	**282**

**Table 2.** Comparison of aquatic invertebrate data obtained by Mark Angelos' ("1993-2004") and our study ("2009-2010"). The numbers represent estimated number of genera. Totals can be found in the bottom-most row of the table. Nomenclature used: "gained" = number of genera recorded in this study, but not by Mark Angelos; "lost" = number of genera recorded by Mark Angelos, but not in this study; "vs." = versus.

**Phylum**	**Class**	**Order**	**1993-2004**	**2009-2010**	**"gained"**	**"lost"**	**Comments**
Mollusca	Gastropoda	<multiple>	5	2	0	3	
Arthropoda	Ostracoda	<multiple>	6	5	0	1	difficult to match: higher taxa vs. species
Arthropoda	Branchiopoda	Anostraca	3	2	0	1	
Arthropoda	Insecta	Ephemeroptera	1	1	0	0	same species
Arthropoda	Insecta	Odonata	8	10	3	1	lost: *Lestes congener*
Arthropoda	Insecta	Hemiptera	7	3	0	4	
Arthropoda	Insecta	Coleoptera	14	6	1	9	
Arthropoda	Insecta	Diptera	24	24	8	8	difficult to match: matures vs. immatures
		**Totals:**	**54**	**44**	**12**	**22**	**net loss = 10 taxa = 19%**

**Table 3.** Summary of order-level statistics of recorded invertebrate taxa. The data is grouped by most salient higher-level taxa. (Sub)totals per higher-level taxon are added in the bottom and top row, highlighted in yellow.

**ePhylum**	Class	Order	**Family**	**taxa**	**Common Name (MMP taxa)**
**8**	**16**	**39**	**222**	**689**	**= Totals**
Annelida	1	2	3	5	Annelids
Arthropoda	8	29	205	669	Arthropods
	Arachnida	3	34	73	Arachnids
		Acari	14	23	Mites
		Araneae	19	48	Spiders
		Solifugae	1	2	Solifugids
	Branchiopoda	2	3	4	Fairy Shrimp, Waterfleas, & kin
		Cladocera	2	2	Waterfleas
		Anostraca	1	2	Fairy Shrimp
	Chilopoda	1	1	1	Centipedes
	Collembola	2	3	6	Springtails
	Insecta	16	157	574	Insects
		Coleoptera	23	106	Beetles
		Dermaptera	2	2	Earwigs
		Diptera	36	155	Flies, Mosquitos, & kin
		Embiidina	1	1	Webspinners
		Ephemeroptera	1	1	Mayflies
		Hemiptera	25	82	True Bugs, Hoppers, Aphids, & kin
		Hymenoptera	27	101	Wasps, Ants, Bees, & Sawflies
		Blattodea	1	1	Termites
		Lepidoptera	20	84	Butterflies & Moths
		Microcoryphia	1	1	Bristletails
		Neuroptera	3	3	Lacewings & Antlions
		Odonata	3	15	Dragonflies & Damselflies
		Orthoptera	5	11	Grasshoppers, Crickets & kin
		Psocoptera	5	7	Barklice
		Thysanoptera	3	3	Thrips
		Zygentoma	1	1	Silverfish
	Malacostraca	3	5	6	Crayfish, Scuds, Isopods, & kin
		Amphipoda	2	3	Scuds
		Decapoda	1	1	Decapods = Crayfish
		Isopoda	2	2	Isopods
	Maxillopoda	1	1	1	Copepods
	Ostracoda	1	1	4	Seed Shrimp
Cnidaria	1	1	1	1	Hydroids
Mollusca	1	2	7	8	Molluscs
	Gastropoda	2	7	8	Snails & Slugs
		Stylommatophora	5	6	Terrestrial Snails & Slugs
		Basommatophora	2	2	Freshwater Snails
Nematoda	2	2	2	2	Nematodes = Roundworms
Platyhelminthes	1	1	2	2	Flatworms
Rotifera	1	1	1	1	Rotifers = Wheel Animals
Tardigrada	1	1	1	1	Tardigrades = Water Bears
**8**	**16**	**39**	**222**	**689**	**=Totals**
