# Supplementary figures and images for: Macro-invertebrate Biodiversity of a Coastal Prairie with Vernal Pool Habitat
Source: Biodivers Data J. 2016 Apr 27;(4):e6732. doi: 10.3897/BDJ.4.e6732 (PMC4867691; doi:10.3897/BDJ.4.e6732)

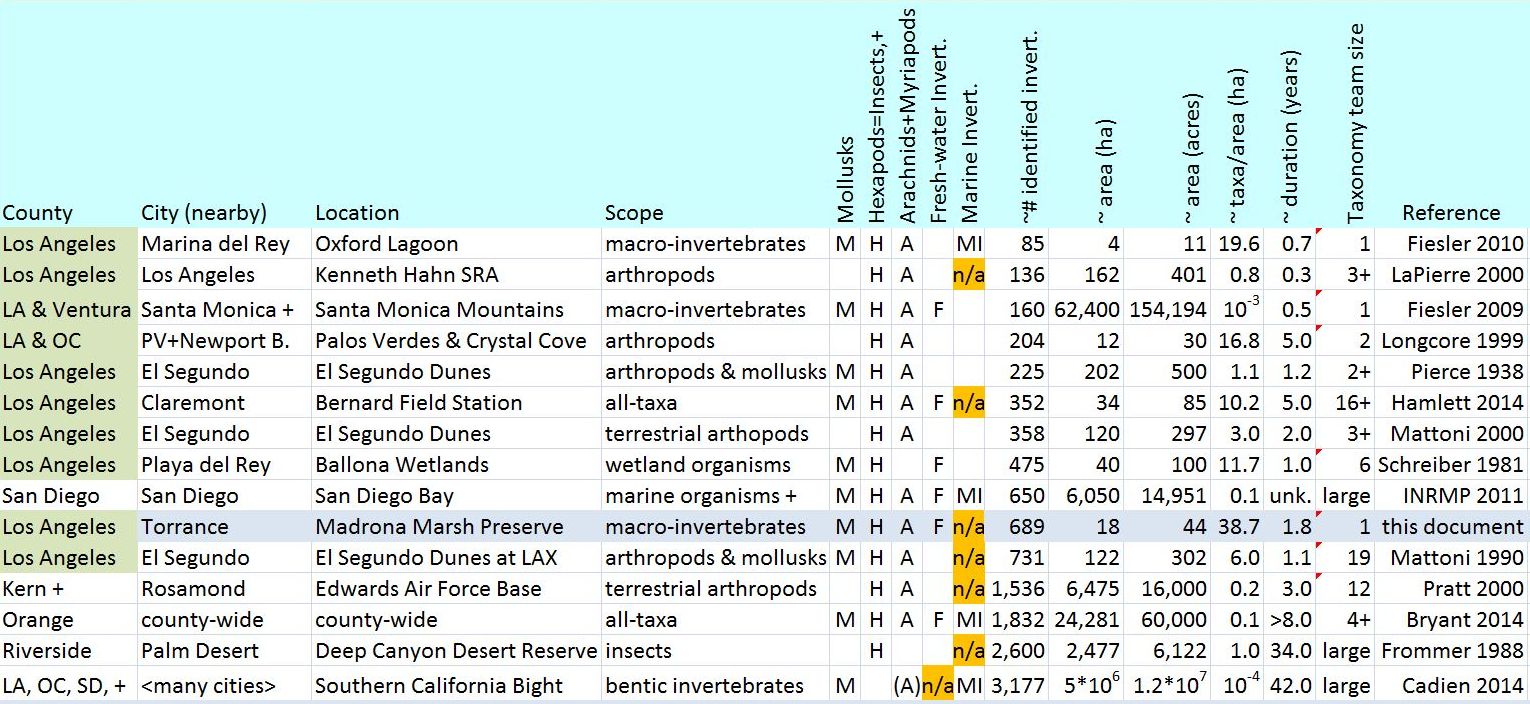

Supplement: Supplementary material 1 — Supplementary file 1 = Selected biological surveys and inventories, that include macro-invertebrates, for Southern California [file biodiversity_data_journal-4-e6732-s001.jpg]
